# Evaluation of Two Amendments (Biochar and Acid Mine Drainage Sludge) on Arsenic Contaminated Soil Using Chemical, Biological, and Ecological Assessments

**DOI:** 10.3390/ma14154111

**Published:** 2021-07-23

**Authors:** Min-Suk Kim, Sang-Hwan Lee, Hyun Park, Jeong-Gyu Kim

**Affiliations:** 1 OJEong Resilience Institute, Korea University, Seoul 02841, Korea; adoniss86@korea.ac.kr; 2 Gyeongin Regional Office, Mine Reclamation Corporation, Seoul 03151, Korea; soillsf@gmail.com; 3 Division of Biotechnology, Korea University, Seoul 02841, Korea; hpark@korea.ac.kr; 4 Division of Environmental Science and Ecological Engineering, Korea University, Seoul 02841, Korea

**Keywords:** dissolved organic carbon, microbial analysis, phytotoxicity, principal component analysis

## Abstract

Various types of organic and inorganic materials are widely examined and applied into the arsenic (As) contaminated soil to stabilize As bioavailability and to enhance soil quality as an amendment. This study deals with two types of amendments: biochar for organic amendment and acid mine drainage sludge (AMDS) for inorganic amendment. Each amendment was applied in two types of As contaminated soils: one showed low contaminated concentration and acid property and the other showed high contaminated concentration and alkali property. In order to comprehensively evaluate the effect of amendments on As contaminated soil, chemical (As bioavailability), biological phytotoxicity (*Lactuca sativa*), soil respiration activity, dehydrogenase activity, urease activity, ß-glucosidase activity, and acid/alkali phosphomonoesterase activity, an ecological (total bacterial cells and total metagenomics DNA at the phylum level) assessment was conducted. Both amendments increased soil pH and dissolved organic carbon (DOC), which changes the bioavailability of As. In reducing phytotoxicity to As, the AMDS was the most effective regardless of soil types. Although soil enzyme activity results were not consistent with amendments types and soil types, bacterial diversity was increased after amendment application in acid soil. In acid soil, the results of principal component analysis represented that AMDS contributes to improve soil quality through the reduction in As bioavailability and the correction of soil pH from acidic to neutral condition, despite the increases in DOC. However, soil DOC had a negative effect on As bioavailability, phytotoxicity and some enzyme activity in alkali soil. Taken together, it is necessary to comprehensively evaluate the interaction of chemical, biological, and ecological properties according to soil pH in the decision-making stages for the selection of appropriate soil restoration material.

## 1. Introduction

A soil amendment is any materials added to soil to improve soil quality and to achieve the goal of improvement. The soil amendments are often used for remediation of arsenic (As) contaminated soil as a stabilizer of As in soil through the formation of insoluble fraction and the decreases in mobility, bioavailability, and toxicity [1]. In the case of an abandoned mine area, the area of contaminated soil is very large and the use of large equipment is often difficult and, thus, it is advantageous to utilize a chemical stabilization method by using amendments [2].

Various types of amendments have been studied, such as silica, clays, and metal oxides [2,3]. Although As mobility and bioavailability are determined by chemical association with different soil solid phases, it is well known that As could be adsorbed on amorphous and crystalline metal (hydro)oxides and co-percipitated on secondary phases with sulfur [4]. Among metals, iron-based materials showed great potential for a chemical stabilization method in As-contaminated soil [3]. The most common form of iron oxides includes ferrihydrite, hematite, goethite, and lepidocrocite and these adsorption sites decreases as a density of adsorption sites are diminished with the crystallization process [5]. In oxidizing conditions such as the environment near an abandoned mine, As is mainly present in the form of arsenate (V) and it binds to iron oxide minerals as an inner-sphere complex such as bidentate and binuclear surface complex [6]. The usefulness of iron in the improvement of As contaminated soil is a well-known fact but because the cost problem of treating a large area is indispensable, studies using by-products containing iron are actively conducted [7]. Lee et al. [8] utilized waste lime stone, red mud, and furnace slag for in situ stabilization of As and reported that an application of iron-based by-products significantly decreased bioavailability and increased microbial activity. Moreover, Kim et al. [9] investigated the applicability of acid mine drainage sludge (AMDS) in trace elements contaminated soil by using chemical and biological assessments and it was reported that AMDS effectively reduced the mobility and bioavailability of trace elements. Byambaa et al. [10] also reported the high removal efficiency of As in a solution using AMDS and revealed the adsorption kinetic through isotherm determination. Although industrial by-products exhibit high efficiency with respect to the immobilization of As, they inevitably contained toxic trace elements, resulting in environmental ecotoxicology risk.

Compared to industrial inorganic amendments, countless raw biomass such as crop residues, forestry residues, food waste, and livestock manure can also be used as an amendment for adsorption and studies by using biochar conversion have been actively investigated in recent decades [11]. Biochar has a porous structure, a large surface area, and a number of functional groups, which provide high adsorption capacity [12]. The adsorption capacity of biochar depended on its various characteristics such as mineral components, pH, pyrolysis condition, etc. [13]. When biochar amended into cationic heavy metal-contaminated soil, heavy metal easily adsorbed onto biochar surface and induced a decrease in the mobility and bioavailability of heavy metal. In the case of As contaminated soil, the application of biochar rapidly reduced arsenate (V) to arsenite (III) and rather increased its mobility. These As species reduction disturb As adsorption due to a negative redox potential [14]. However, biochar application could reduce As uptake by maize due to Ad adsorption onto the biochar surface [15]. In addition, Kim et al. [16] reported that there were changes in As availability by spent coffee ground biochar in acid, neutral, and alkali soils. Dissolved organic carbon released from biochar, phosphorus, and As are in a competitive adsorption relationship with one another, which is also a major challenge in demonstrating the mechanisms of As behavior in soil system [6]. Therefore, these findings have cause difficulty when trying to apply biochar in As contaminated soil.

Since the amendment in contaminated soil is irreversible after being applied once, the selection of an appropriate amendment will be a very important decision-making step [17]. In order to evaluate the effect of amendment on the environment as a whole, a number of studies have been investigated in parallel with biological assessment as well as chemical assessment [9,18,19]. Many types of single extraction and sequential extraction were used for chemical assessment and toxicity tests using terrestrial plant and small animal were also utilized for biological assessment [8,16,20]. In recent years, as part of ecological risk assessment, in order to evaluate the ecological function of the soil, microbial community, and its health, the decomposers are also determined [21]. However, it is also true that studies considering the ecological part are relatively less conducted because it is difficult to select a reference sample and more time is required [22]. In particular, studies that have conducted multiple assessment in the evaluation and selection process of amendment are insufficient.

Thus, the objective of this study was to comprehensively evaluate the effect of different types of amendment materials on soil characteristics with respect to microbial analysis, enzyme activity tests, phytotoxicity tests for biological assessments, and several chemical assessments and to derive major factors for the amelioration process of contaminated soil.

## 2. Materials and Methods

### 2.1. Preparation of Soil and Amendments

Two types of As contaminated soils were collected at an abandoned mine area in Gangwon Province, Republic of Korea. After air-drying, soil samples were sieved though a 2 mm seive. Two types of amendments were used in this study. For inorganic amendment, AMDS was collected from acid mine drainage treatment facility in Gangwon Province, Republic of Korea. The collected wet sample was dried at 110 °C in a dry-oven for 48 h and passed through a 0.5 mm sieve. The pH and electrical conductivity of AMDS are 8.36 and 0.76 ds m^−1^, respectively [16]. Among the trace elements, only Cd (30 mg kg^−1^), Pb (6 mg kg^−1^), and Zn (966 mg kg^−1^) were detected [16]. The contents of free metal oxides (Al, Fe, and Mn) extracted by ammonium oxalate buffer were 379.05, 25.67, and 1.04 g kg^−1^, respectively [16]. In order to evaluate the leaching possibility from AMDS, Korea standard leaching test (KSLT) was conducted [23] and the results showed that only Pb is detected at 0.01 mg L^−1^ (legal standard is 3 mg L^−1^) [16]. For organic amendment, spent coffee grounds were collected from a nearby commercial coffee shop. A dried sample was pyrolyzed at 400 °C for 30 min in an electrical furnace to produce spent coffee grounds char (SCGC). The manufactured SCGC was also passed through a 0.5 mm sieve. Each amendment was added in each soil with 2% ratio based on each weight and homogenized. The treated soil samples were aged, with maintenance of soil moisture at 60% of the water holding capacity of each soil for 4 weeks.

### 2.2. Analysis of Soil Physico-Chemical Properties and Amendments

The total two types of soil samples were prepared and pH and electrical conductivity (EC) of soils were determined using pH-EC meter (Thermo Orion 920A, Waltham, MA, USA) after shaking in a soil:water (5 g: 25 mL) suspension solution. Loss on ignition (LOI) was determined at 40 °C over 16 h [24]. Particle distribution and soil texture was determined based on the pipette method according to Stokes law [25]. The dissolved organic carbon (DOC) was determined after shaking soil:water (1 g: 10 mL) suspension solution and filtration (0.45 μm) by using an automatic total organic carbon analyzer (Shimadzu, TOC-VCPH, Tokyo, Japan) [19]. The total As concentration was analyzed according to ISO 11,466 [26] by using an aqua regia solution (HCl:HNO_3_ = 3:1, *v/v*). The bioavailability of As was determined according to the Esnaola and Millan [27] procedure extracted with 0.5 M CaCl_2_ solution. The fraction of As in soil was assessed by using Wenzel’s sequential extraction procedure [28]. The As concentration in extracted solution was quantified by using induced plasma coupled optical emission spectrophotometry (ICP-OES, Agilent, Santa Clara, CA, USA). The accuracy of the analytical data with regards to the As was assessed using certified reference material (NIST 2711a, Montana II Soil, Gaithersburg, MD, USA). In addition, the pH and total As concentration of two amendments were also determined in the same methods as the soil analysis method.

### 2.3. Phytotoxicity Test and Enzyme Activity Test for Biological Assessment

In order to ensure the effect of soil amendments and its impacts on plant development, lettuce (*Lactuca sativa* L.) was cultivated with controlled conditions. Twelve seeds were placed in petri dishes containing 40 g of treated soil. After germination, one or two seedlings were removed from the dishes to unify the number of seedlings per dish into 10. The moisture content of dish was maintained at approximately 60% of soil water holding capacity. All dishes were placed in a controlled growth chamber (16 h for daylight and 8 h for night, 20 ± 3 °C) and cultivated for 2 weeks. After cultivation, all seedlings were harvested, washed with deionized water, and its length was immediately measured by using desktop scanner (V700, Epson, Suwa, Japan) and an image analyzer program (WinRhizo 5.0a, Reagent). After that, the fresh weight of seedlings was determined and dried in an oven at 70 °C. Dried seedling samples were digested with HNO_3_ and H_2_O_2_ using a microwave to measure the amount of As uptake. The As concentration in filtrate was quantified by using ICP-OES and the accuracy of the As measurement was performed by using certified reference material (BCR (Community Bureau of Reference)-402, white clover, Brussels, Belgium).

Soil respiration activity (SRA) was measured by using the CO2 trap method using NaOH solution in closed jar [29]. Dehydrogenase activity (DHA) was assayed by the reduction in 2,3,5-triphenyltetrazolium chloride (TTC) to triphenylformazan (TPF) [30]. Urease activity (URA) was assayed by a reduction in urea to ammonium ion [31]. Acid phosphatase activity (ACP), alkaline phosphatase activity (ALP), and ß-glucosidase activity (GLU) was assayed using modified universal buffer at each optimal pH, each accurate substrate, and the determination of ρ-nitrophenol [32]. All enzyme activities were determined using a spectrophotometer (Shimadzu, UV-1650PC).

### 2.4. Microbial Analysis for Simplified Ecological Assessment

#### 2.4.1. Microbial Counts

The quantification of the total bacterial cells present in the non-inoculated samples was performed by tuf qPCR. This quantitative PCR targets the tuf gene, which has been shown to be highly conserved among a large number of bacterial genera, mainly as a single copy in their genome [33]. A quantitative tuf qPCR kit for bacteria (Takara Bio, Inc., Otsu, Shiga, Japan), using SYBR Green technology, was used. The reaction was performed with 5 μL of template DNA in a total volume of 25 μL, according to the manufacturer’s protocol (Takara Bio). All amplifications were run on an Agilent AriaMax Real-Time PCR system (Agilent). A negative control (ultrapure water instead of sample DNA) was included in each run. All amplification reactions were run in triplicate.

#### 2.4.2. DNA Extraction, Sequencing, and Microbial Community Analysis

The total metagenomic DNA was extracted and purified using the DNeasy PowerSoil Pro Kit (Qiagen, Valencia, CA, USA) according to the manufacturer’s instructions. Final DNA extracts were subject to electrophoresis in 1% agarose gel and quantified by the NanoDrop 2000 (Thermo Fisher Scientific, Waltham, MA, USA). In order to produce a 16S amplicon with Pacbio, genomic DNA was amplified using KAPA HiFi HotStart Ready Mix PCR kit (Cat No. KK2660) according to the protocol provided by Pacific Biosciences (Menlo Park CA, USA). High Fidelity (HiFi) reads of 16S rRNA genes were generated by the SMRT^®^ Link software (v.3.1.1) with circular consensus sequences (CCS) mode. In order to demultiplex sequences, we applied barcode sequences of each sample to the lima, which is designed for PacBio data. Microbial community analysis was processed using QIIME v2018.6 [34]. Demultiplexed fastq reads were imported into QIIME by using the CASAVA 1.8 format for paired-end sequences. Chimeric sequences, marginal sequence errors, and noisy sequences were filtered by using DADA2 [35]. Dereplicated sequences were further clustered into operational taxonomic units using the GREENGENES database at 97% similarity while employing the VSEARCH open reference OTU picking technique [36]. The clustered sequences were assigned taxonomies using q2-feature-classifier plugin [37] and the Naïve Bayes classifier that was trained on the Greengenes (May 2013 release) 99% OTUs. Alpha and Beta diversity analysis was performed in R using the Phyloseq package [38]. 

### 2.5. Data Analysis

All of the determinations were performed in triplicate for each sample. One-way analysis of variance (one-way ANOVA) was used to compare the means of the different treatments. Where significant *p*-values (*p* < 0.05) were obtained, the differences between the means were evaluated using Tukey’s test. The relationships among the experimental results were evaluated using Pearson correlation analysis and principal component analysis (PCA). The first two principal components (PC) were plotted on a two-dimensional plane. The data were analyzed using the SAS program (SAS 9.4, SAS Institute Inc., Cary, NC, USA).

## 3. Results and Discussion

### 3.1. Basic Soil Characteristics

The pH values were different among the two types of soil (Table 1). Soil A and B presented slightly acid and alkali properties, respectively. Organic contents were similar in two soils. The silt and clay contents were higher in soil B than soil A. Since soils were collected near the abandoned mine area, both soils were highly contaminated with As (754 and 2999 mg kg^−1^ of soil A and B, respectively) and the concentrations were much higher than domestic soil regulation (50 and 150 mg kg^−1^ is the worrisome level and countermeasure level, respectively, for forest and land soil). By using the sequential extraction process, five types of As fraction were determined with high recovery efficiency in both soils. The percentage of the sum of fraction 1, fraction 2, and fraction 3 relative to the total amount indicated mobility factor (MF) [39,40] considered the potential risk of As in soil. The MF index of soil A and B are 62.8% and 74.7%, respectively, which represented that the extent of As contamination and that the impact on the ecosystem could be concerning. The pH of SCGC and AMDS were 6.9 and 8.0, respectively, and As was not detected in both amendments.

### 3.2. Effect of Amendment Materials on Soil Characteristics 

In soil A, both amendments significantly increased soil pH and DOC (Table 2). It was well known that the application of biochar induced protonation and supplement of basic cation, resulting in the increase in soil pH [41,42]. After the application of biochar into the soil, the biochar acts as a sink of carbon sequestration and a source of DOC releases into the soil environment [43]. The increased soil pH also facilitated a degradation of biochar into dissolved form, resulting in the increases in DOC in AMDS treatment though low organic content of AMDS (Table 2). Kim et al. [18] also reported that increases in soil pH by the application of organic matter and lime affected the DOC concentration. Thus, both SCGC and AMDS increased DOC contents. The increased DOC could enhance the bioavailability of trace elements in soil through the formation of metal-DOC complexes [44]. By observing these results, it could be supposed that bioavailable As could be increased after amendments application. However, the bioavailable As was decreased by SCGC and AMDS treatment and this is observed significantly in soil A (Table 2). This result implied that the stabilization effects by the adsorption of As onto biochar and metal oxide surface acts more than the effect of increased bioavailability by DOC [45]. In case of soil B, only AMDS increased soil pH, significantly. The pH of control soil showed 8.30, which indicated slightly alkali conditions. This suggested that the effect of protonation and the basic cation supply by SCGC were not sufficient to increase soil pH more than 8.30. On the other hand, AMDS could increase soil pH over 8.45 and the pH increase by AMDS in alkaline soil was also observed in previous studies [16,45]. The As adsorption and stabilization effects of AMDS containing high concentration of metal oxides and a large surface area were sufficiently exhibited even in alkali conditions [46], but SCGC significantly increased bioavailability of As on the contrary. Chen et al. [47] also reported similar results to this study that biochar treatment shows a contrasting effect on the retention of phosphorous (P) in acid and alkali soil condition due to the enhanced transport of metal oxides-associated P in soil solution. Considering that the chemical structure and behavior property in soil of As and P are similar, the bioavailability of As increased in SCGC treatment with relatively few metal oxides compared to the AMDS treatment under alkali condition. These results suggested that research to discover the threshold of soil pH that determines the adsorption of As in soil by metal oxides and desorption by DOC us required.

### 3.3. Effect of Amendments on Lettuce Growth and Biological Properties

**Figure 1:**
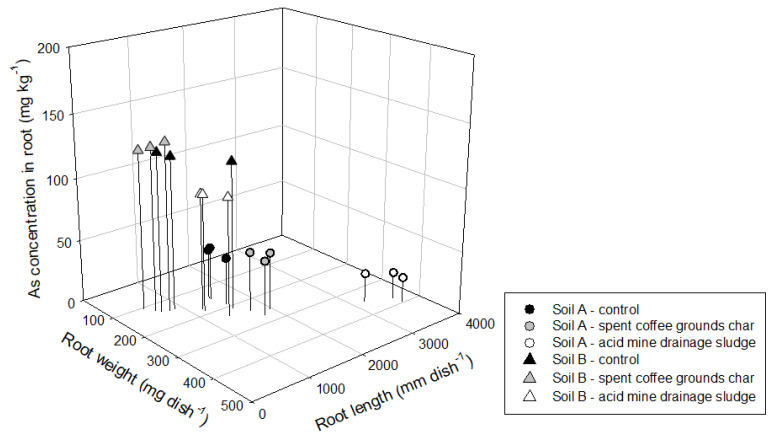
Distribution of weight, length, and As concentration in lettuce root after 4 weeks cultivation.

A cultivation experiment using lettuce was conducted to confirm the effect of changes in chemical properties of acid and alkali soil by amendment treatment on organisms and the length, fresh weight, and As concentration of root that is in direct contact with the soil were analyzed (Figure 1). In soil A, root length of lettuce was highest in ADMS treatment as 3502 mm dish^−1^, lowest in control as 1519 mm dish^−1^, and the medium in SCGC (1.814 mm dish^−1^). In soil B, root length was also highest in ADMS treatment as 1249 mm dish^−1^, lowest in SCGC as 647 mm dish^−1^, and medium in control (1041 mm dish^−1^). The trend of the root fresh weight result was also similar to the root length. The bioavailable content of As could be easily absorbed by plant root, resulting in the expression of phytotoxicity through lipid peroxidation and cell death by the overproduction of reactive oxygen species at levels exceeding the intrinsic antioxidant capacity [48,49]. Therefore, the results of the concentration of As in the lettuce root showed that the bioavailability of As was well reflected, showing the lowest concentration in AMDS (white circle and triangle).

**Figure 2:**
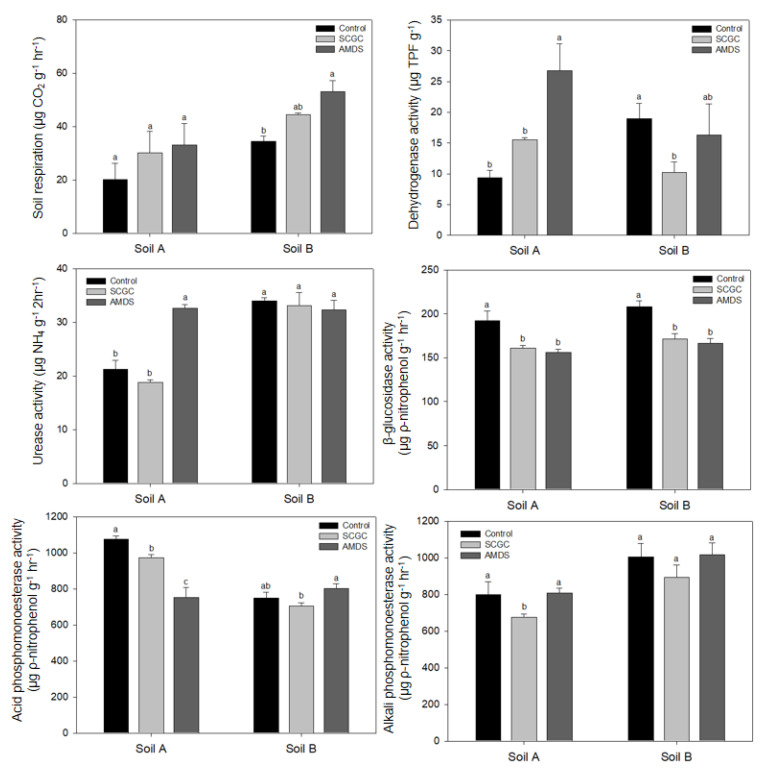
Effect of amendments on soil biological properties. Different letters indicate the significant differences at the 5% level according to Tukey’s test.

In addition to chemical analysis, the results of soil enzyme activity could provide useful information on As toxicity with the respect to soil quality [19]. Considering the nutrient cycle in soil ecosystem, five types of soil enzyme activities and soil respiration by microbe were assessed (Figure 2). Soil respiration activity (SRA) represented the total aerobic microbe activity and showed increasing trends by amendment treatment in soil A and B. Although there was no dramatic change due to the amendments, it could be observed that the overall biological quality increased. In soil A, amendment treatment increased DHA and indicated the increase in soil quality. However, in soil B, it was hard to find any significant amelioration in soil quality. In the case of URA, only AMDS in soil A could increase enzyme activity significantly. Since URA was easily inhibited in alkali condition by ammonium ion, there were changes in URA in soil B [50]. Kim et al. [45] also reported that URA has negative relationship with soil pH by using multiple regression analysis in alkali soil. In the case of GLU, both amendments decreased GLU in both soils. This is because, similar to URA, GLU decreased as soil pH increased from 4.5 to 8.5 [51]. However, Koo et al. [19] revealed that GLU has a negative relationship with bioavailable As concentration in mine tailings. On the contrary, GLU seems to be more dependent on soil pH than bioavailable As. Phosphomonoesterases were also important extracellular enzymes in the P cycle in soil environment, which mineralizes organic-P into inorganic-P species [52]. Since the optimum pH range for ACP is 4 to 6.5, an increase in soil pH by amendment treatment in soil A decreased ACP significantly. For the same reason, it was difficult to confirm a significant change in ACP by amendment in alkaline soil B and the value of ACP was all lower than that of soil A. Moreover, ALP was also sensitive to changes in soil pH and the value of ALP is higher in soil B than soil A [53]. Instead, there was a tendency of a partial decrease in SCGC treatment in both soils. When the microbial activity results were summarized, it was observed that most of the soil pH was greatly affected and some differences were found in soil B, which has relatively low pH changes.

### 3.4. Effect of Amendments on Microbial Activity and Diversity

**Figure 3:**
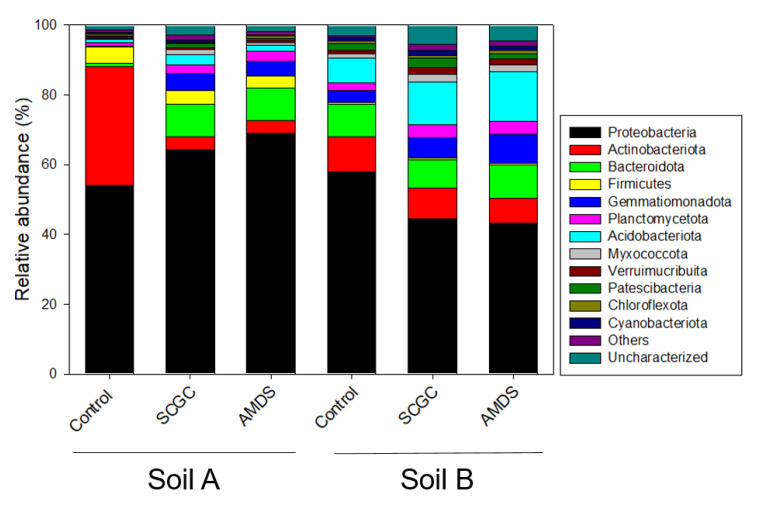
Comparison of the relative abundance of microbial consortium at the phylum level.

Changes in the number of bacterial OTU by amendments were confirmed and their richness and diversity were also calculated (Table 3). By observing the changes in the number of OTU and Chao 1 index, both amendments had positive effect on bacterial richness in both soil and the differences in terms of the type of amendment were not large. Aanderud et al. [54] reported that the changes in the number of OTU and bacterial richness caused by snowfall work together. Therefore, it was supposed that the increase in the richness and OTU caused by the amendment was linked to an increase in the total amount of microorganisms and was expressed as an increase in SRA (Figure 2). Proteobacteria is a dominant phylum in all soils and treatments (Figure 3). Although the soil bacteria community had been influenced by several environmental conditions, Proteobacteria was a dominant phylum that was easily observed in many soil environments such as forest, agricultural, and coastal areas [55,56]. The application of amendments in As contaminated soil increased not only bacteria richness but also its diversity (Table 3). Figure 3 also represented the relative abundance of microbial consortium at the phylum level in soil. The diversity of soil A was very low and, thus, the two phyla, which are Proteobacteria and Actinobacteriota accounted, for 90% of the total. However, the application of amendments decreased an abundance of Actinobacteriota and increased the proportion of other phylum, resulting in an increase in overall diversity. Acidophilic Actinobacteriota was common in terrestrial habitats such as forest and mine soil in the pH range from 3.5 to 6.5, such as soil A [57]. Therefore, the application of amendment increased soil pH from acidic to neutral and alkaline, which decreases Actinobacteriota abundance. As the abundance of Actinobacteriota decreased, the abundance of the other phylum relatively increased. Although the changes in soil microbial diversity by amendment treatment in soil B was smaller than that of soil A, it was found that the abundance of individual phylum increased by amendment treatment. Increasing the abundance of the individual phylum could be seen as having resistance and resilience to disturbance in the future, which in turn could mean an improvement in overall soil quality [58].

### 3.5. Principal Component Analysis and Major Soil Factors

**Figure 4:**
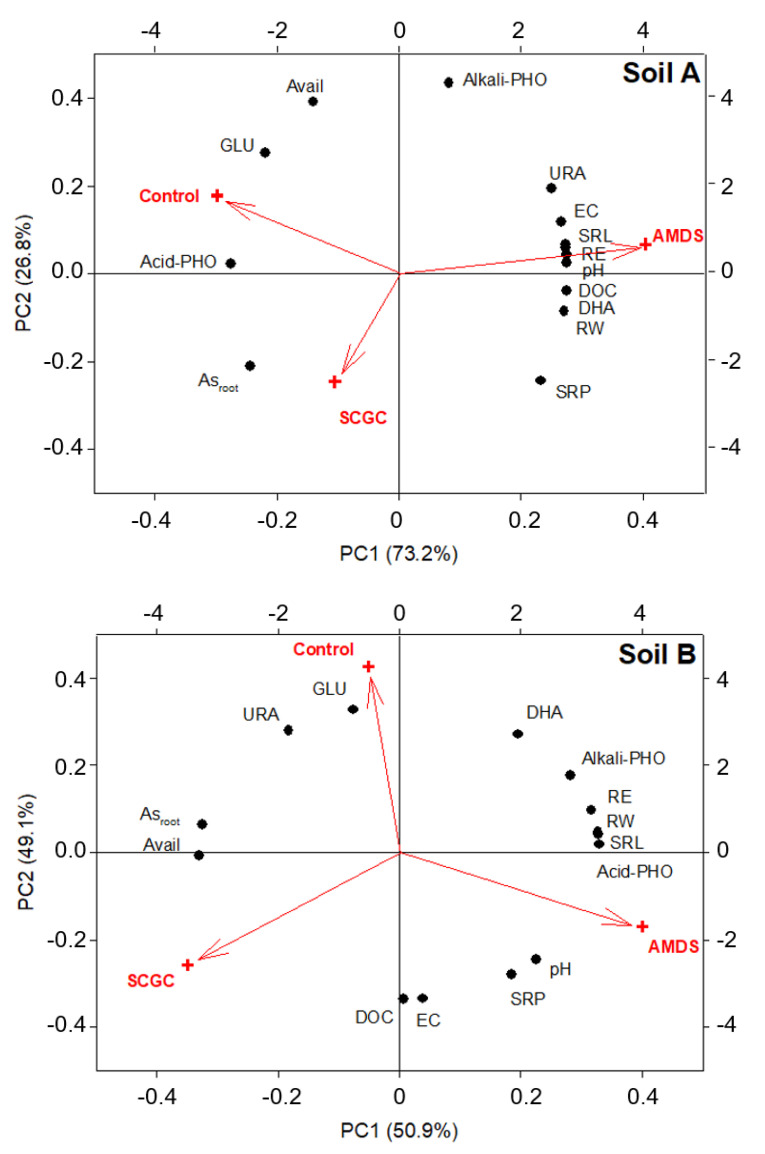
Distribution of the soil properties (EC, electrical conductivity; DOC, dissolved organic carbon; Avail, bioavailability of As), phytotoxicity results (root length; root weight; RootAs, As concentration in root), soil biological properties (SRA, soil respiration activity; DHA, dehydrogenase activity; URA, urease activity; GLU, ß-glucosidase activity; Acid-PHO, acid phosphatase activity; Alkali-PHO, alkali phosphatase activity), and ecological properties (OTU, operational taxanomic unit; shannon, simpson) of two soils plotted against the first and second principal component (PC) from principal component analysis.

PCA was conducted to confirm the relationships among the experimental results. These results showed that PC1 explained 72.3% and 54.9% of the variance and PC2 explained 27.7% and 45.1% in soil A and B, respectively. For soil A, the factors with the largest absolute eigen vectors in PC1 and PC2 were root weight, DHA, SRP, GLU and OTU, and ALP, As in root, URA, available As, and simpson, respectively. For soil B, the factors with the largest absolute eigen vectors in PC1 and PC2 were URA, SRP, GLU, pH and Shannon, and root length, Chao1, root weight, ACP, and DHA, respectively. In the case of soil A, Figure 4 showed that AMDS treatment located opposite to the control with respect to the x-axis was positively correlated with plant growth, soil enzyme activities (URA, DHA, and SRP), and ecological properties (OTU, Shannon, and Simpson). In addition, the above indicators were located opposite to As bioavailability. That is, the characteristics of contaminated soil (control) could be explained by the As bioavailability and it could be interpreted that there was a positive improvement in the above indicators due to the decrease in As bioavailability by AMDS treatment. Ghosh et al. [20] revealed that bioavailable As exerted greater inhibitory effect on microbial population and DHA by using linear regression analysis. In addition, Koo et al. [19] also revealed that water soluble As was the most important factor relative to soil enzyme activity (DHA and GLU) using PCA and multiple regression analysis. By using chemical assessment, the application of amendments increased DOC significantly in soil A (Table 2). It could be supposed that increased DOC enhances As bioavailability, resulting in an inhibition of plant growth and soil enzyme activity. Contrary to expectations, the DOC was located in the same dimensional plane as biological and ecological properties (Figure 4). These results indicated that the reduction in the As bioavailability by amendments and the correction of soil pH from acidic to neutral had a greater effect on improving soil quality than the adverse effect of DOC increased by increasing pH. Although dissolved organic matter played a vital role in the As mobility in soil and groundwater system, it was judged that its role was relatively small when it was not originated in the natural equilibrium state but by an amendment introduced from an external system [59]. These results suggested that AMDS application seems to be an effective chemical amendment for As stabilization in acid soil condition despite of the slight increases in DOC.

In the case of soil B, where the pH change by the amendment was not as large as soil A, it was difficult to identify the factors that clearly explain the effect of the amendment on soil quality improvement. The plotted factors were largely grouped into two groups: one contains DHA, ACP, ALP, root length, and root growth and the other included pH, EC, DOC, SRA, OTU, Shannon, and Simpson (Figure 4). In particular, by considering the fact that the ecological properties were located on opposite sides of the control for both x, and y axes, the ecological property seems to ameliorate through amendment treatment. However, given that As bioavailability was closer to SCGC and As concentration in lettuce root in the dimensional plane, it is suggested that the increase in As bioavailability by the increases in DOC derived from biochar might have a negative effect under alkaline soil condition. Figure 4 showed that DOC had a positive relationship with ecological properties but was located on opposite sides of the plant growth and several soil enzyme activities. Therefore, DOC still had an environmental risk and should be carefully observed as a monitoring factor, especially in alkaline soil condition. On the one hand, to ameliorate these side effects of DOC in alkali soil contaminated with As, Kim et al. [45] proposed the cultivation of green manure in term of phytostabilization technology. By using cultivation, it was confirmed that the root zone effect expressed in specific root length had a positive effect on SRA, URA, and DHA as well as DOC using stepwise multiple regression. In addition, the co-application of iron-based material also ameliorated the side effect of DOC derived from organic matter in soil [60]. Prior to the proposal of a general treatment plan, a scientific and quantitative evaluation of the impact factors and the integration of chemical, biological, and ecological assessments should be conducted in parallel. Furthermore, due to the pore structure, which is also affected by earthworms and the physical properties of the soil that are related to important biogeochemical processes such as carbon turnover, nitrate transformation, and As transport [61,62,63], pilot scale experiments in the field seem to be required when considering micro and macro pores. 

## 4. Conclusions

In this study, the difference in the effect of amendment materials according to the soil pH was studied in a complex manner by using chemical, biological, and ecological assessment for As contaminated soil. In case of acid soil, AMDS was effective in terms of soil pH correction from acid to neutral and As stabilization due to supply adsorption sites. The effect was reflected in biological and ecological assessments. However, in alkali soil, the effect of AMDS was not sufficiently exerted as compared to acid soil due to the complexity of the behavior of DOC. In the selection process of the restoration material and post-monitoring stage, biological and ecological assessments seem to be considered for an integrated ecological risk assessment.

## Figures and Tables

**Table 1:** Physico-chemical properties of experiment soils.

Soil Property	Soil A	Soil B
pH	5.8 ± 0.3	8.3 ± 0.3
EC ^1^ (us cm^−1^)	35 ± 2.6	75 ± 1.5
LOI ^2^ (%)	4.9 ± 0.3	4.5 ± 0.1
Sand (%)	80.3 ± 1.2	64.6 ± 1.1
Silt (%)	2.5 ± 0.1	11.2 ± 3.0
Clay (%)	17.2 ± 0.6	24.2 ± 2.2
Soil texture ^3^	sandy loam	sandy clay loam
Total As (mg kg^−1^)	784 ± 12	2999 ± 124
Wenzel Sequential extraction ^4^		
Fraction 1 (mg kg^−1^)	2.4 ± 0.4	46.5 ± 1.3
Fraction 2 (mg kg^−1^)	107.2 ± 2.7	262.8 ± 11.0
Fraction 3 (mg kg^−1^)	395.8 ± 6.4	1931.4 ± 129.0
Fraction 4 (mg kg^−1^)	221.3 ± 10.7	523.8 ± 92.6
Fraction 5 (mg kg^−1^)	78.2 ± 3.2	234.0 ± 12.4
Sum	804.9	2998.4
Recovery (%)	102.6	99.9

^1^ Electrical conductivity; ^2^ Loss-on-ignition; ^3^ Soil texture was classified according to the USDA triangle; ^4^ Arsenic fractionation was measured by using Wenzel’s sequential extraction. Fraction 1, non-specifically sorbed; Fraction 2, specifically sorbed; Fraction 3, amorphous and poorly crystalline hydrous metal oxides; Fraction 4, well crystallized hydrous metal oxides; Fraction 5, residual.

**Table 2:** Effect of amendments on chemical properties of soils *.

Treatment		pH	EC ^1^	DOC ^2^	Extractable As ^3^
Soil A	Control	5.78 ± 0.03 c	35 ± 2.6 b	109.0 ± 5.3 c	0.14 ± 0.01 a
	SCGC ^4^	6.20 ± 0.05 b	36 ± 2.1 b	126.2 ± 0.1 b	0.06 ± 0.00 b
	AMDS ^5^	8.06 ± 0.02 a	111 ± 3.6 a	186.5 ± 6.4 a	0.08 ± 0.02 b
Soil B	Control	8.30 ± 0.03 b	75 ± 1.5 b	136.5 ± 3.5 b	1.57 ± 0.06 b
	SCGC	8.36 ± 0.06 ab	119 ± 4.2 a	184.6 ± 7.2 a	1.91 ± 0.01 a
	AMDS	8.45 ± 0.03 a	119 ± 6.0 a	179.6 ± 9.9 a	1.08 ± 0.04 c

* Different letters means significant difference among treatments. ^1^ Electrical conductivity (us m^−1^); ^2^ Dissolved organic carbon (mg kg^−1^); ^3^ Ca(NO_3_)_2_ extractable As concentration (mg kg^−1^); ^4^ Spent coffee grounds char; ^5^ Acid mine drainage sludge.

**Table 3:** Number of operational taxonomic unit (OUT), estimated OUT richness (Chao 1), Shannon and Simpson diversity indices, and estimated sample coverage (Good’s coverage).

Treatment		OTU	Chao 1	Shannon	Simpson	Good’s Coverage
Soil A	Control	2299	14,349.01	7.538	0.9692	0.7974
	SCGC ^1^	3138	9827.65	10.038	0.9968	0.7702
	AMDS ^2^	3263	10,061.11	9.763	0.9914	0.7566
Soil B	Control	4265	15,885.57	10.259	0.9951	0.6642
	SCGC	5451	18,111.69	11.667	0.9992	0.5879
	AMDS	5366	16,197.01	11.705	0.9993	0.6059

^1^ Spent coffee grounds char; ^2^ Acid mine drainage sludge.

## Data Availability

The data presented in this study are available upon request from the corresponding author.
